# The Modern Surgical Approach to Pulmonary Atresia with Ventricular Septal Defect and Major Aortopulmonary Collateral Arteries

**DOI:** 10.3390/children9040515

**Published:** 2022-04-05

**Authors:** Matteo Trezzi, Enrico Cetrano, Sonia B. Albanese, Luca Borro, Aurelio Secinaro, Adriano Carotti

**Affiliations:** 1 Units of Cardiac Surgery, Department of Cardiac Surgery, Cardiology, Heart and Lung Transplantation, Bambino Gesù Children’s Hospital IRCCS, 00165 Rome, Italy; matteo.trezzi@opbg.net (M.T.); enrico.cetrano@opbg.net (E.C.); sonia.albanese@opbg.net (S.B.A.); 2 Advanced Cardiothoracic Imaging Unit, Department of Imaging, Bambino Gesù Children’s Hospital IRCCS, 00165 Rome, Italy; luca.borro@opbg.net (L.B.); aurelio.secinaro@opbg.net (A.S.)

**Keywords:** Fallot’s tetralogy, pulmonary atresia with ventricular septal defect, major aortopulmonary collateral arteries, unifocalization, rehabilitation

## Abstract

Pulmonary atresia with ventricular septal defect and major aortopulmonary collaterals is a complex congenital heart defect that includes a heterogeneous subgroup of patients. Variation in the sources of pulmonary blood flow contributes to the complexity of the lesion and the diversity of approaches to its management. Unifocalization and rehabilitation focus on mobilization of collateral arteries and growth of native pulmonary arteries, respectively, with the ultimate surgical goal of achieving separated systemic and pulmonary circulations with the lowest possible right ventricular pressure. Regardless of the strategy, outcomes have altered the natural history of the disease, with a complete repair rate of approximately 80% and low early and late mortality rates. Given this heterogeneity of pulmonary vasculature, a tailored approach should be adopted for each patient, using all diagnostic methods currently offered by technical developments.

## 1. Introduction

Pulmonary atresia with ventricular septal defect and major aortopulmonary collateral arteries (PA/VSD/MAPCAs) is a complex congenital heart defect that includes a heterogeneous subgroup of patients characterized by different pulmonary perfusion patterns, of which MAPCAs are an important, although not the only source. True pulmonary arteries may have varying degrees of hypoplasia, be absent and, more rarely, discontinuous, with unilateral pulmonary perfusion provided by the arterial duct and contralateral by MAPCAs [1,2]. The variability of the pulmonary perfusion pattern is a determining factor in the complexity of the lesion and the consequent diversity of its surgical management.

The ideal end goal of treatment is to establish pulmonary blood flow from the right ventricle (RV) to as normal a pulmonary vascular bed as possible to allow VSD closure with the lowest possible RV pressure after repair. In order to pursue this aim, however, a structured approach based on a thorough understanding of the anatomical characteristics of each patient is required. 

## 2. Preoperative Imaging 

At our center, every patient with PA/VSD/MAPCAs first receives cardiopulmonary imaging by computed tomography (CT). Given its non-invasiveness, CT is suitable for diagnosis in the neonatal period and allows to decide at the patient’s initial presentation which surgical procedure should be considered, i.e., either unifocalization or pulmonary circulation rehabilitation. In most patients, treatment of the disease in the neonatal period is not required, and the diagnosis is supplemented by cardiac catheterization in the vicinity of surgery. Cardiac catheterization plays a key role in the preoperative evaluation and is considered an essential step. The purpose of cardiac catheterization is to identify the sources of pulmonary blood flow; the presence, size, and confluence of the central pulmonary arteries and their branches in the lungs; areas of stenosis in the lobar or segmental branches; and the origin and course of the MAPCAs and their connections to the true pulmonary arteries. In particular it should be emphasized the key role of the pulmonary vein wedge injection for the demonstration of native pulmonary arteries not detectable with other methods and its consequent central role in the surgical planning of this peculiar disease. In late referrals, which are common, especially in humanitarian cases, cardiac magnetic resonance (CMR) imaging now plays an important role in the diagnosis and surgical planning of patients with PA/VSD/MAPCAs. In addition, CT scan and CMR are the main sources of advanced 3D technology that we are increasingly using in clinical practice.

### 2.1. Computed Tomography

CT is a reliable panoramic tomography modality for less invasive assessment of comprehensive cardiothoracic anatomy in patients with PA/VSD/MAPCAs [3,4,5,6,7]. CT is particularly accurate in isolating true pulmonary arteries and detecting small collateral arteries. In particular, it is sometimes considered superior to cardiac catheterization in defining the mediastinal relationship between the great vessels, airways, and esophagus, and it has demonstrated accuracy in assessing complex anatomic overlaps that would necessarily be difficult to define with conventional two-dimensional imaging techniques. We use it as the second imaging technique after 2D echocardiography to determine the type of surgical procedure the patient should undergo and as a primary source for 3D technologies.

### 2.2. Magnetic Resonance Imaging

CMR imaging provides also functional information for surgical decision making in patients with PA/VSD/MAPCAs. In our practice, CMR is a particularly useful adjunct to cardiac catheterization, either in late referrals or in staging when it is critical to assess total pulmonary blood flow and Qp:Qs ratio in view of possible complete delayed repair of the disease. Indeed, in this disease, systemic blood flow (Qs) can be measured as the addition of superior venous caval flow and descending aortic flow (distal to the origin of MAPCAs). Pulmonary blood flow (Qp) can be measured either as total pulmonary venous return [8,9,10] or, in the presence of pulmonary atresia, by subtracting Qs from ascending aortic flow [11]. The preoperative Qp:Qs ratio calculated with CMR has been shown to correlate with RV systolic pressure after VSD closure, proving useful in selecting patients for complete repair [11]. Finally, coronal isotropic contrast-enhanced magnetic resonance angiography (CEMRA) with a gadolinium bolus tracking technique can be applied to obtain a 3-dimensional angiographic dataset.

### 2.3. D Reconstructions of CT or CMR Images

3D technology in cardiac surgery is now a consolidated clinical practice that has proven over the years that it can improve the surgical approach in complex clinical cases [12]. In our center, starting from the images of CT, but also from CMR sequences when available, we routinely use 3D technology in patients with PA/VSD/MAPCAs. In addition to traditional 3D volume rendering reconstructions, we increasingly use virtual anatomical models of the patient’s mediastinal and pulmonary anatomy [13]. From a purely technical point of view, the procedure for 3D reconstruction of an anatomical model of PA/VSD/ MAPCAs is divided into 3 main phases:

(1) Acquisition of CT images;
(2) 3D image segmentation using dedicated 3D medical software;
(3) Processing and editing of the virtual three-dimensional anatomical model.

Virtual models allow the surgeon to view and navigate the screen using a mouse, trackball, or remote control and can be used for surgical planning both preoperatively and intraoperatively. They allow the spatial relationship of MAPCAs to the aorta, airways, esophagus, and pulmonary veins to be identified. In addition, they allow determination of the exact origin and course of the collaterals in relation to the main bronchi and their branches and precise surgical planning of unifocalization preoperatively. Such preoperative planning can shorten surgical time and lead to improved clinical outcomes for the patient.

## 3. Surgical Treatment 

The goals of surgical treatment are to achieve a single confluence of all sources of pulmonary blood flow (unifocalization) and to complete the repair by closing the VSD and creating a connection between the RV and pulmonary artery (PA). Originally, these aims were pursued in multiple stages over years [14], but now repair can be performed even in a single operation in infancy [15]. Both the single- and multistage approaches have resulted in different outcomes in terms of mortality and achievement of complete repair. The choice of surgical approach for these patients depends mainly on the institutional policy, although unifocalization of MAPCAs appears to be somehow the best choice for recruiting as many lung segments as possible. Initial management and timing of intervention in infants with PA/VSD/MAPCAs is determined by the degree of pulmonary blood flow, according to which patients can be classified into 3 groups: (1) infants with cyanosis and no evidence of heart failure (stenotic MAPCAs or underdeveloped native pulmonary arteries); (2) infants with heart failure with large MAPCAs; and (3) balanced circulation infants, when pulmonary blood flow through the MAPCAs and native pulmonary arteries is just sufficient to have mild cyanosis and no heart failure.

### 3.1. Rehabilitation 

Pulmonary artery rehabilitation aimed at inducing native pulmonary artery growth was the first surgical treatment described for PA/VSD/MAPCAs. Gates-Laks central shunt [16] is probably the most commonly performed procedure, although some groups still prefer RV connection to the pulmonary artery. The Melbourne group [17,18] advocates a strict rehabilitation policy supporting an early approach with the central shunt, followed by reassessment of pulmonary artery growth, conversion to an RV-to-pulmonary artery conduit, and hopefully complete repair with or without augmentation of the pulmonary artery branches. However, this approach relies almost exclusively on the presence of native pulmonary arteries. Another strategy for PA rehabilitation is stenting of the ductus arteriosus supplying a discontinuous pulmonary artery (usually the left). 

**Figure 1:**
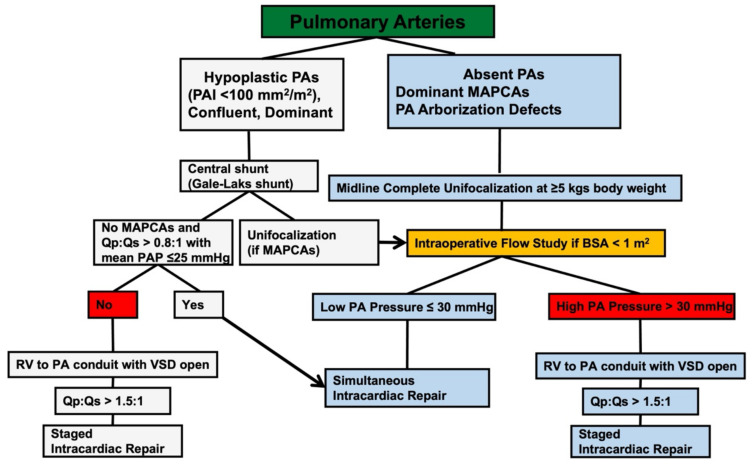
Childrens’ Hospital Bambino Gesù algorithm for PA/VSD/MAPCAs treatment in infancy according to the presence, size and distribution of native pulmonary arteries.

Our current technique of choice for rehabilitation is the central shunt. We perform rehabilitation not only in patients with small confluent branch pulmonary arteries with normal arborization and dual-supply collaterals, but also in patients with prevalent nonexclusive distribution of native pulmonary arteries to the lungs (see Section 3.2, Figure 1), in whom unifocalization can be performed as a second step procedure [19]. In all patients who are clinically stable after rehabilitation, cardiac catheterization is performed several months after the procedure, and depending on the results, we may proceed with unifocalization alone, in the case of associated terminal MAPCAs, unifocalization and repair, or just repair. The decision whether to perform a concomitant repair after unifocalization is based on the intraoperative pulmonary flow study (see Section 3.3). 

### 3.2. Unifocalization 

The unifocalization technique, particularly in single-stage constitutes the approach of choice advocated by the Stanford group [20]. Access to the mediastinum during one-stage unifocalization is achieved via midline sternotomy. All collaterals originating from the descending thoracic aorta and/or from the epiaortic vessels are dissected, separated from their origin and anastomosed to each other and/or to the native pulmonary arteries to create a new pulmonary vascular tree that is adequately expanded through the use of homograft tissue. At this point, possibly after pulmonary pressure testing (see Section 3.3), the VSD is closed and the RV is connected to the unifocalized pulmonary vascular tree with a valved homograft conduit. In a subset of patients unsuitable for VSD closure, palliation is performed with shunt placement on the unifocalized pulmonary arteries [21]. Early removal of MAPCAs from the systemic circulation prevents both MAPCA degeneration and native pulmonary artery regression. Therefore, early unifocalization provides vascularization of all healthy pulmonary segments using both native pulmonary arteries and collaterals as source material. 

Our institutional approach for PA/VSD/MAPCAs is similar to that of the Stanford group. Pulmonary arterial rehabilitation is performed in all patients with confluent hypoplastic (i.e., pulmonary arterial index less than 100 mm^2^/m^2^) [22] but dominant (i.e., distributed over most pulmonary segments) pulmonary arteries with eventual MAPCAs supplying the areas not perfused by the true pulmonary arteries (Figure 1).

Unifocalization and rehabilitation focus on mobilization of collateral arteries and growth of native pulmonary vessels, respectively. Regardless of the strategy, the results have altered the natural history of the disease, with a complete cure rate of approximately 80% and low early and late mortality. In our experience to date, the ability to achieve definitive intracardiac repair is the critical factor for improved survival and adequate systolic RV performance at midterm follow-up [19]. Patients with the most unfavorable anatomy (absent central pulmonary arteries and hypoplastic MAPCAs) remain a challenge.

### 3.3. The Role of the Intraoperative Flow-Study in Assessing the Feasibility of Concomitant VSD Closure 

The use of the intraoperative flow study as a functional measure of pulmonary vascular performance and suitability for VSD closure was first reported by Reddy and colleagues [23]. In October 1996 our group adopted the intraoperative flow study to assess the feasibility of VSD closure at the time of unifocalization and since then it has been performed according to an unchanged standardized protocol. The neopulmonary arterial confluence is cannulated to be perfused via a line derived from the arterial port of the oxygenator, and a pressure recording is placed simultaneously. The test is started with lungs deflated and the heart beating and fully vented. By a stepwise 25% increase, a maximum flow of oxygenated blood equal to 2.5 L · min^−1^ · m^−2^ is pumped into the reconstructed pulmonary bed and the mean pulmonary artery pressure (mPAP) is simultaneously recorded. An mPAP value of 30 mm Hg or less is selected for VSD closure, predicting adequate postoperative RV pressure. On the other hand, if mPAP rises above 30 mm Hg during the incremental steps of the flow study, the test is terminated, intracardiac repair is not performed and either a RV to PA conduit or a central shunt is placed based on individual patient assessment.

Proper performance of an intraoperative flow study can be problematic in patients with a body surface area greater than 1 m^2^ because excessive priming of the pump is required and the ability of the pump oxygenator to maintain adequate both systemic and pulmonary blood flow is compromised. Late presenting humanitarian referrals encompassing more than 1 m^2^ of body surface area are subject to preoperative CMR imaging with the Qp:Qs ratio calculation and VSD closure decided accordingly if the Qp:Qs value exceeds 1.5:1.

### 3.4. Criteria for Delayed VSD Closure

At our institution, patients who did not receive VSD closure in the first instance are re-evaluated, also on the basis of the individual clinical condition, 6–12 months after surgery by cardiac catheterization, possibly associated with CMR. Intracardiac repair is indicated in the presence of Qp:Qs greater than 1.5, if their source of pulmonary blood flow is an RV to PA connection, or in the presence of Qp:Qs greater than 0.8 and mPAP less than 25 mmHg, if their source of pulmonary blood flow is a systemic-to-pulmonary shunt.

## 4. Bambino Gesù Children’s Hospital Results

**Figure 2:**
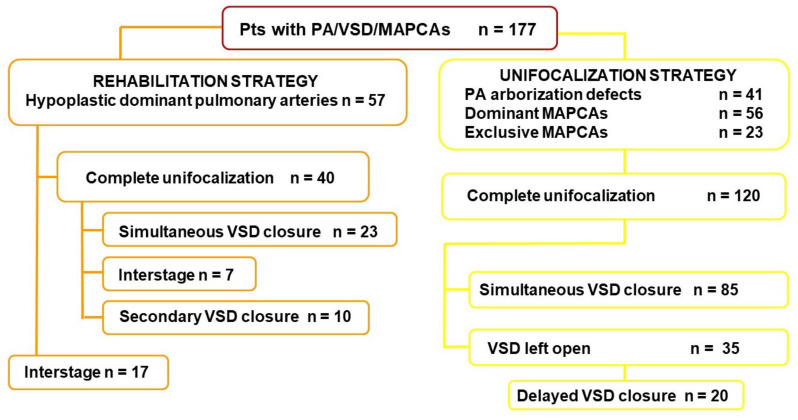
Surgical results of PA/VSD/MAPCAs patients treated at Bambino Gesù Children Hospital.

**Figure 3:**
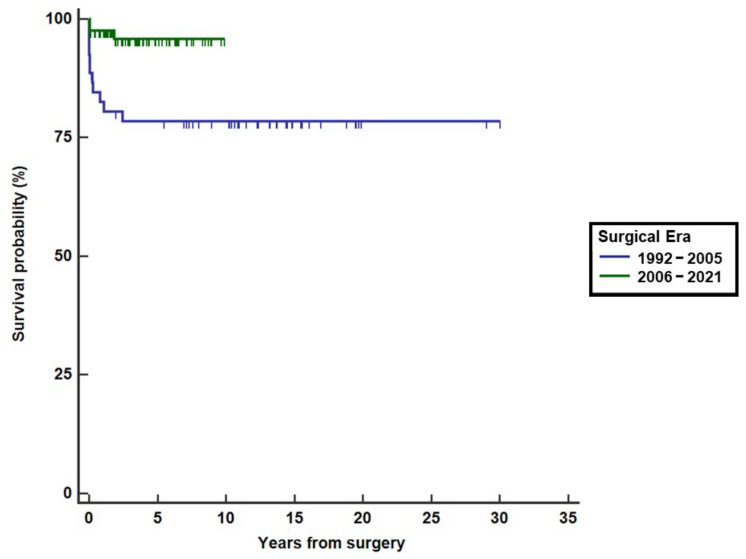
Survival by the era of all treated patients with PA/VSD/MAPCAs.

Based on the above considerations and previously reported experience [24], from January 1994 to November 2021 we treated 177 patients with PA/VSD/MAPCAs (Figure 2), with a mean age of 12 months (range, 10 days–35 years) at the first procedure. Since the beginning of our experience, survival has improved significantly, reflecting both the surgical learning curve and improvement in postoperative medical care, as shown in Figure 3 (log-rank, *p* = 0.001), and is close to 100% in recent years. Prior to 2005, mortality was influenced by 22q11 deletion syndrome (hazard ratio 6.3, 95% CI 1.9–20.2), whereas in recent years it no longer played a significant role (log-rank, *p* = 0.4). Based on a recent analysis, the overall rate of complete intracardiac repair in our series was approximately 83% for all patients who underwent unifocalization, either primarily or after rehabilitation procedures [25,26]. Survivors were followed-up for a median follow-up of 6 years (range, 0.01–30.1). Their hemodynamic outcome was shown to be stable over time, as evidenced by a mean RVSP/SBP ratio (ratio of RV systolic pressure to systolic blood pressure) of 0.45 ± 0.14 measured in the subgroup of patients who underwent cardiac catheterization after biventricular repair (either primary or staged) at a median interval of 95 months after repair. The intraoperative pulmonary vascular compliance test played a key role in selecting patients suitable for simultaneous intracardiac repair after unifocalization, using a mean pulmonary artery pressure cutoff of 30 mm Hg.

## 5. Clinical Cases

**Figure 4:**
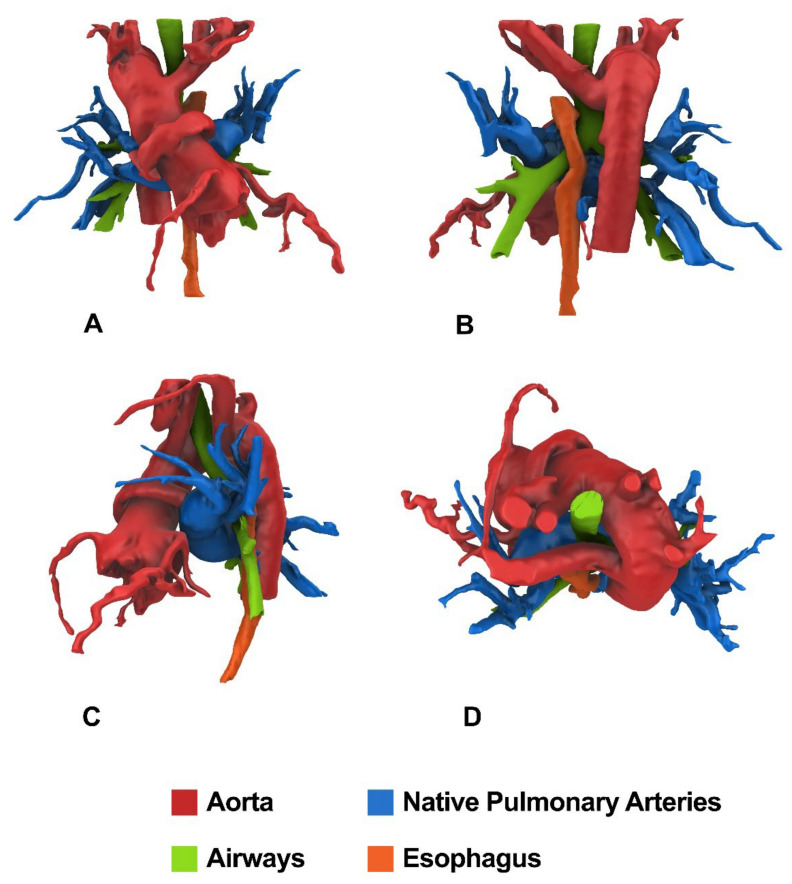
CT imaging of the patient who underwent pulmonary artery rehabilitation and subsequent unifocalization with a central shunt. Colored rendering for Frontal (**A**), Posterior (**B**), Lateral (**C**), and Top (**D**) views obtained from CT images with semi-automatic 3D segmentation process.

Patient # 1. An 8 months/7.6 kg male with PA/VSD/MAPCAs (5 collaterals) and confluent but hypoplastic intrapericardial pulmonary arteries underwent pulmonary artery rehabilitation and subsequent unifocalization with a central shunt. Figure 4 shows CT anatomy after unifocalization. 

**Figure 5:**
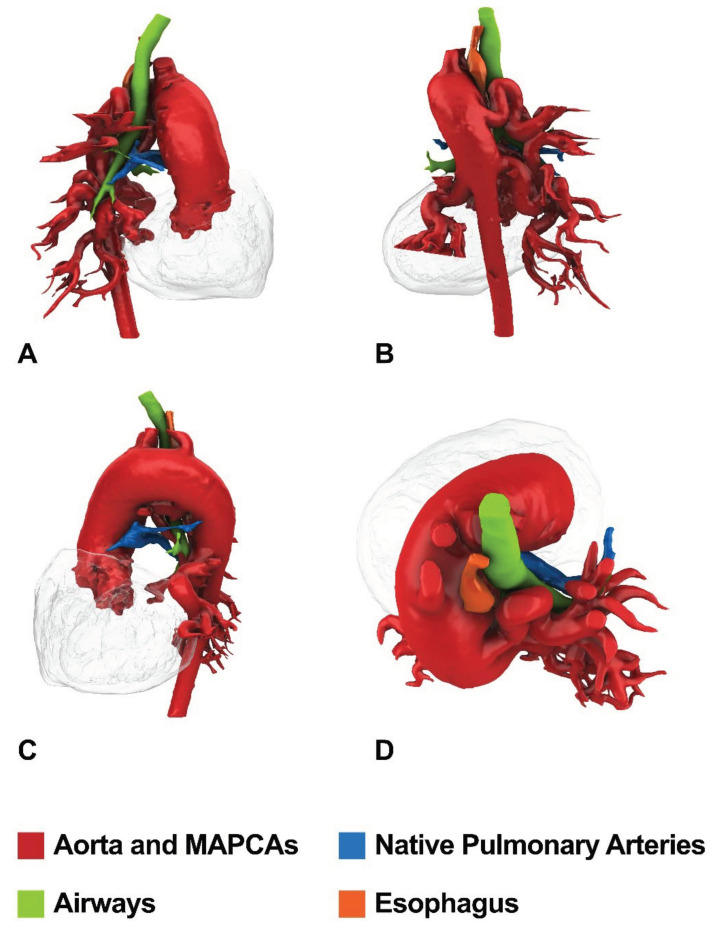
Preoperative CT imaging of the patient who underwent one-stage unifocalization and repair. Colored rendering for Frontal (**A**), Posterior (**B**), Lateral (**C**), and Top (**D**) views obtained from CT images with semi-automatic 3D segmentation process.

Patient # 2. A 7-year-old/15.8 kg (late referral) male underwent one-stage repair by unifocalization of all collaterals, reconstruction of the new pulmonary confluence, and VSD closure (intraoperative flow-study pressure of 20 mm Hg). Figure 5 shows the preoperative CT anatomy.

## 6. Conclusions

The modern approach to PA/VSD/MAPCAs is the synthesis of decades of experience of the world’s leading centers in the treatment of this disease. The experience gained at our institution confirms that a structured approach to the treatment of this complex lesion is the key to achieve optimal results. In particular, over the course of the years we have demonstrated how staged and single-stage approaches should not be considered separate philosophies, but should be used as part of an integrated approach, based on rigorous patient selection. In this sense, both rehabilitation and unifocalization strategies, possibly used in sequence where feasible, play a fundamental role. From this perspective, modern diagnostic methods and 3D technology are an important tool for preoperative diagnosis and surgical planning. The ultimate goal is to achieve definitive intracardiac repair which has proved to be the main determinant of both improved survival and adequate RV systolic performance during mid-term follow-up. 

## Data Availability

The data presented in this study are available on request from the corresponding author. The data are not publicly available due to privacy concerns.

## References

[1] Jefferson K., Rees S., Somerville J. (1972). Systemic arterial supply to the lungs in pulmonary atresia and its relation to pulmonary artery development. Heart.

[2] Rabinovitch M., Herrera-Deleon V., Castaneda A.R., Reid L. (1981). Growth and development of the pulmonary vascular bed in patients with tetralogy of Fallot with or without pulmonary atresia. Circulation.

[3] Ryan J.R., Moe T.G., Richardson R., Frakes D.H., Nigro J.J., Pophal S. (2015). A Novel Approach to Neonatal Management of Tetralogy of Fallot, With Pulmonary Atresia, and Multiple Aortopulmonary Collaterals. JACC Cardiovasc. Imaging.

[4] Liu J., Li H., Liu Z., Wu Q., Xu Y. (2016). Complete Preoperative Evaluation of Pulmonary Atresia with Ventricular Septal Defect with Multi-Detector Computed Tomography. PLoS ONE.

[5] Secinaro A., Curione D. (2019). Congenital heart disease in children. Medical Radiology—Diagnostic Imaging.

[6] Ciancarella P., Ciliberti P., Santangelo T.P., Secchi F., Stagnaro N., Secinaro A. (2020). Noninvasive imaging of congenital cardiovascular defects. Radiol. Med..

[7] Secinaro A., Curione D., Mortensen K.H., Santangelo T.P., Ciancarella P., Napolitano C., Del Pasqua A., Taylor A.M., Ciliberti P. (2019). Dual-source computed tomography coronary artery imaging in children. Pediatr. Radiol..

[8] Malayeri A.A., Spevak P.J., Zimmerman S.L. (2015). Utility of a High-Resolution 3D MRI Sequence (3D-SPACE) for Evaluation of Congenital Heart Disease. Pediatr. Cardiol..

[9] Niu J., Profirovic J., Pan H., Vaiskunaite R., Voyno-Yasenetskaya T. (2003). G protein βγ subunits stimulate p114RhoGEF, a guanine nucleotide exchange factor for RhoA and Rac1: Regulation of cell shape and reactive oxygen species production. Circ. Res..

[10] Schicchi N., Secinaro A., Muscogiuri G., Ciliberti P., Leonardi B., Santangelo T.P., Napolitano C., Agliata G., Basile M.C., Guidi F. (2015). Multicenter review: Role of cardiovascular magnetic resonance in diagnostic evaluation, pre-procedural planning and follow-up for patients with congenital heart disease. Radiol. Med..

[11] Grosse-Wortmann L., Yoo S.-J., Van Arsdell G., Chetan D., Macdonald C., Benson L., Honjo O. (2013). Preoperative total pulmonary blood flow predicts right ventricular pressure in patients early after complete repair of tetralogy of Fallot and pulmonary atresia with major aortopulmonary collateral arteries. J. Thorac. Cardiovasc. Surg..

[12] Tack P., Victor J., Gemmel P., Annemans L. (2016). 3D-printing techniques in a medical setting: A systematic literature review. Biomed. Eng. Online.

[13] Byrne N., Forte M.V., Tandon A., Valverde I., Hussain T. (2016). A systematic review of image segmentation methodology, used in the additive manufacture of patient-specific 3D printed models of the cardiovascular system. JRSM Cardiovasc. Dis..

[14] Rome J.J., E Mayer J., Castaneda A.R., E Lock J. (1993). Tetralogy of Fallot with pulmonary atresia. Rehabilitation of diminutive pulmonary arteries. Circulation.

[15] Reddy V.M., McElhinney D.B., Amin Z., Moore P., Parry A.J., Teitel D.F., Hanley F.L. (2000). Early and Intermediate Outcomes After Repair of Pulmonary Atresia With Ventricular Septal Defect and Major Aortopulmonary Collateral Arteries. Circulation.

[16] Gates R.N., Laks H., Johnson K. (1998). Side-to-Side Aorto–Gore-Tex Central Shunt. Ann. Thorac. Surg..

[17] Mumtaz M.A., Rosenthal G., Qureshi A., Prieto L., Preminger T., Lorber R., Latson L., Duncan B.W. (2008). Melbourne Shunt Promotes Growth of Diminutive Central Pulmonary Arteries in Patients With Pulmonary Atresia, Ventricular Septal Defect, and Systemic-to-Pulmonary Collateral Arteries. Ann. Thorac. Surg..

[18] D’Udekem Y., Alphonso N., Nørgaard M.A., Cochrane A.D., Grigg L.E., Wilkinson J.L., Brizard C.P. (2005). Pulmonary atresia with ventricular septal defects and major aortopulmonary collateral arteries: Unifocalization brings no long-term benefits. J. Thorac. Cardiovasc. Surg..

[19] Carotti A., Albanese S., Filippelli S., Ravà L., Guccione P., Pongiglione G., Di Donato R.M. (2010). Determinants of outcome after surgical treatment of pulmonary atresia with ventricular septal defect and major aortopulmonary collateral arteries. J. Thorac. Cardiovasc. Surg..

[20] Reddy V., Liddicoat J.R., Hanley F.L. (1995). Midline one-stage complete unifocalization and repair of pulmonary atresia with ventricular septal defect and major aortopulmonary collaterals. J. Thorac. Cardiovasc. Surg..

[21] Mainwaring R.D., Patrick W.L., Roth S.J., Kamra K., Wise-Faberowski L., Palmon M., Hanley F.L. (2018). Surgical algorithm and results for repair of pulmonary atresia with ventricular septal defect and major aortopulmonary collaterals. J. Thorac. Cardiovasc. Surg..

[22] Itatani K., Miyaji K., Nakahata Y., Ohara K., Takamoto S., Ishii M. (2011). The lower limit of the pulmonary artery index for the extracardiac Fontan circulation. J. Thorac. Cardiovasc. Surg..

[23] Reddy V., Petrossian E., McElhinney D.B., Moore P., Teitel D.F., Hanley F.L. (1997). One-stage complete unifocalization in infants: When should the ventricular septal defect be closed?. J. Thorac. Cardiovasc. Surg..

[24] Carotti A. (2019). Surgical Management of Fallot’s Tetralogy With Pulmonary Atresia and Major Aortopulmonary Collateral Arteries: Multistage Versus One-Stage Repair. World J. Pediatr. Congenit. Heart Surg..

[25] Trezzi M., Albanese S.B., Albano A., Rinelli G., D’Anna C., Polito A., Cetrano E., Carotti A. (2017). Impact of Pulmonary Flow Study Pressure on Outcomes After One-Stage Unifocalization. Ann. Thorac. Surg..

[26] Trezzi M., D’Anna C., Rinelli G., Brancaccio G., Cetrano E., Albanese S., Carotti A. (2018). Midterm Echocardiographic Assessment of Right Ventricular Function After Midline Unifocalization. Ann. Thorac. Surg..

